# Clockwise Anterior-to-Posterior—Double Isolation (CAP-DI) Approach for Portal Lymphadenectomy in Biliary Tract Cancer: Technique, Yield, and Outcomes

**DOI:** 10.3390/cancers14235770

**Published:** 2022-11-24

**Authors:** Andrew J. Sinnamon, Eric Luo, Aileen Xu, Sarah Zhu, Jason W. Denbo, Jason B. Fleming, Daniel A. Anaya

**Affiliations:** 1 Department of Gastrointestinal Oncology, H. Lee Moffitt Cancer Center & Research Institute, Tampa, FL 33612, USA; 2 College of Medicine, University of South Florida Morsani, Tampa, FL 33612, USA

**Keywords:** portal lymphadenectomy, yield, lymph node, outcomes, technique

## Abstract

**Simple Summary:**

Cancers can arise from the liver bile duct system and gallbladder, known as cholangiocarcinomas and gallbladder cancers. When surgery is possible to remove the cancer, removing the lymph nodes that provide drainage (the portal lymph nodes) is important for determining the correct stage and providing important prognostic information. However, removal of these lymph nodes can be technically challenging for surgeons. The goal of this study was to evaluate postoperative outcomes after a reproducible approach to surgical removal of these lymph nodes. A stepwise, illustrated description of the technique is provided. Typically, surgeons aim to remove at least six lymph nodes when performing this operation. However, it was found that this does not always occur despite best efforts. Fewer lymph nodes were removed in older patients, which might be related to biology. Removal of the lymph nodes added time to surgery when compared to patients who did not have portal lymph nodes removed for other reasons, but did not result in worse outcomes for patients.

**Abstract:**

Background: Portal lymphadenectomy (PLND) is the current standard for oncologic resection of biliary tract cancers (BTCs). However, published data show it is performed infrequently and often yields less than the recommended 6 lymph nodes. We sought to identify yield and outcomes using a Clockwise Anterior-to-Posterior technique with Double Isolation of critical structures (CAP-DI) for PLND. Methods: Consecutive patients undergoing complete PLND for BTCs using CAP-DI technique were identified (2015–2021). Lymph node (LN) yield and predictors of LN count were examined. Secondary outcomes included intraoperative and postoperative outcomes, which were compared to patients having hepatectomy without PLND. Results: In total, 534 patients were included; 71 with complete PLND (36 gallbladder cancers, 24 intrahepatic cholangiocarcinomas, 11 perihilar cholangiocarcinomas) and 463 in the control group. The median PLND yield was 5 (IQR 3–8; range 0–17) and 46% had at least 6 nodes retrieved. Older age was associated with lower likelihood of ≥6 node PLND yield (*p* = 0.032), which remained significant in bivariate analyses with other covariates (*p* < 0.05). After adjustment for operative factors, performance of complete PLND was independently associated with longer operative time (+46.4 min, *p* = 0.001), but no differences were observed in intraoperative or postoperative outcomes compared to the control group (*p* > 0.05). Conclusions: Yield following PLND frequently falls below the recommended minimum threshold of 6 nodes despite a standardized stepwise approach to complete clearance. Older age may be weakly associated with lower PLND yield. While all efforts should be made for complete node retrieval, failure to obtain 6 nodes may be an unrealistic metric of surgical quality.

## 1. Introduction

Portal lymphadenectomy (PLND) for examination of the periportal regional nodal basin is advocated for biliary tract cancers (BTCs). Specifically, the 8th edition of the AJCC staging manual recommends evaluation of a minimum of 6 lymph nodes when performing PLND for a gallbladder or intrahepatic cholangiocarcinoma (ICC), while lymphadenectomy without a specified nodal count is recommended for perihilar cholangiocarcinoma and distal extrahepatic cholangiocarcinoma [1,2]. These recommendations are drawn from the fact that the rate of nodal metastases in these diseases are relatively common—reaching up to 60% based on T-stage and primary site [3,4,5,6,7,8]—and that prognostication is improved when more nodes are sampled [9,10,11,12]. However, while national data shows that rates of PLND appear to be increasing over time, its use remains underutilized, with up to 40–50% of patients never having PLND at the time of liver resection for gallbladder cancer or ICC [13,14,15]. Furthermore, when PLND is performed, it has been reported that the recommended 6 lymph nodes are only retrieved approximately 25% percent of the time or less frequently, with median lymph node count yield ranging from 1–2 (registry data) to 3–6 (high-volume specialized centers) [4,5,10,11,12,14,15,16,17,18]. In fact, the benchmark of 6 lymph nodes was derived from a large single-institution study including 122 patients, in which retrieval of 6 lymph nodes or more was associated with more accurate staging, but for which the median yield was only 3 lymph nodes [10]. With the lack of granularity in national data, there remains some uncertainty as to whether this is due to differences in surgical techniques for PLND, pathologic count, or a real reflection of the number of nodes contained within the lymphatic basin.

**Figure 1:**
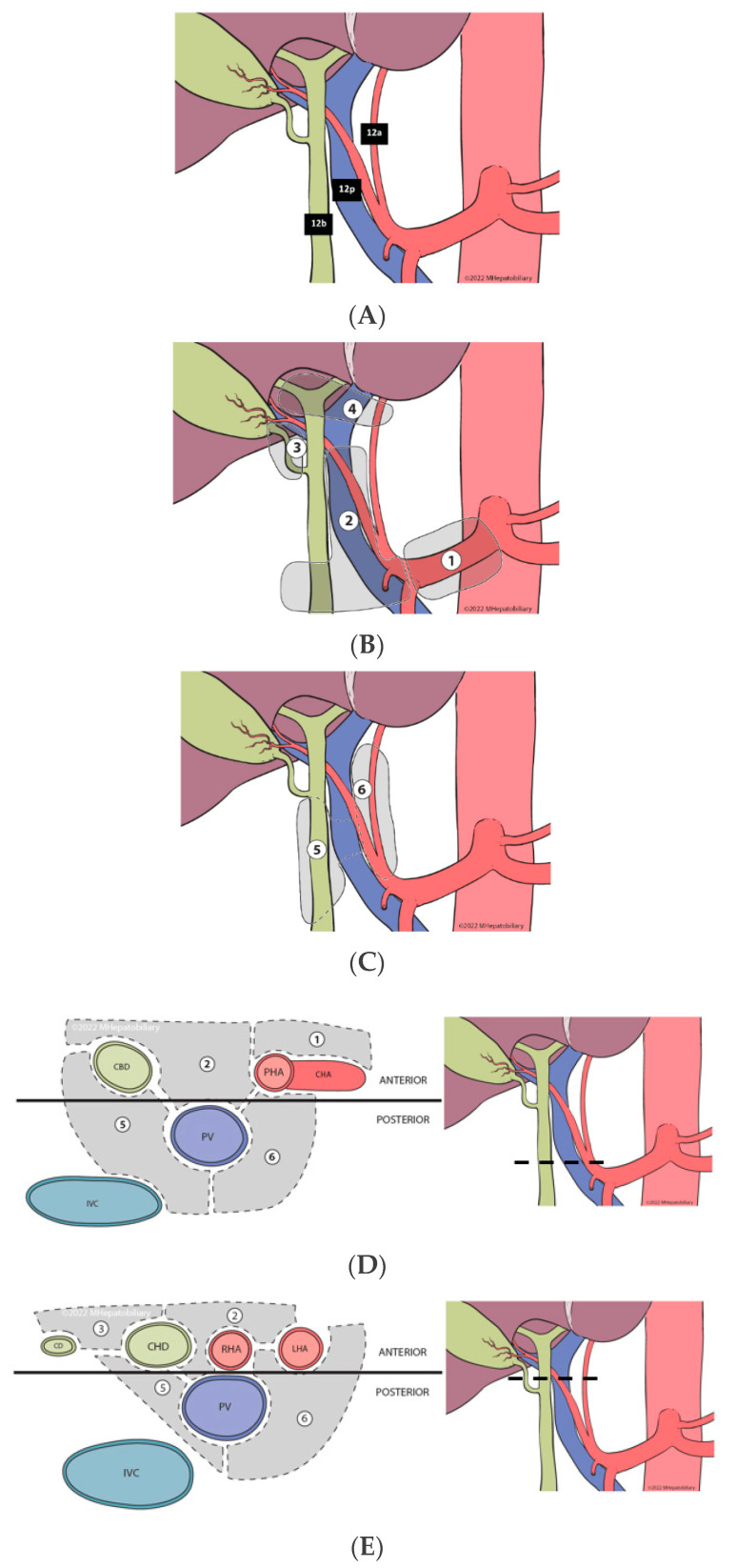
(**A**). Anatomy of intra-abdominal nodal stations defined by the Japanese gastric cancer designation. (**B**). Localization of anterior lymphatic substations of the hepatoduodenal ligament—coronal plane. (**C**). Localization of posterior lymphatic substations of the hepatoduodenal ligament and their relation to the portal vein—coronal plane. (**D**). Axial view of the most caudal portion of the hepatoduodenal ligament—with designation of anterior and posterior lymphatic substations—divided by a coronal line across the anterior surface of the portal vein. (**E**). Axial view of the most cephalad portion of the hepatoduodenal ligament—with designation of anterior and posterior lymphatic substations—divided by a coronal line across the anterior surface of the portal vein.

Despite the rising call for adherence to guidelines for accurate nodal staging of BTCs, there are few published descriptions of the technical aspects of PLND as a reference for practicing surgeons and/or trainees, and a formal standardized approach for PLND is lacking. Ultimately, the goal of PLND is regional clearance of all the fibrofatty lymph-containing tissue surrounding the hepatoduodenal ligament, and those corresponding to stations 12a, 12b and 12p, under the Japanese gastric cancer lymph node designations (Figure 1A) [19]. As such, judicious dissection of the node-containing tissue within this region is important for optimizing lymph node yield, while avoiding injury to the hepatic artery (HA), common bile duct (CBD) and portal vein (PV).

In this context, we herein describe a reproducible, step-wise technique for PLND performed in a ***C***lockwise, ***A***nterior-to-***P***osterior fashion with ***D***ouble ***I***solation of critical structures (***CAP-DI***). Our goal was to examine and characterize lymph node yield following this standardized technique in a cohort of consecutive patients with biliary tract cancers undergoing curative-intent resection, and to critically assess and compare this yield to the current benchmark of 6 or more lymph nodes. Lastly, we sought to evaluate the impact of a complete PLND by comparing intraoperative and postoperative outcomes of PLND in contrast to a comparison group of patients having hepatectomy without PLND.

## 2. Methods

### 2.1. Study Cohort

All consecutive patients with liver malignancies (primary and metastatic) undergoing curative-intent hepatectomy at Moffitt Cancer Center, were identified from a prospective institutional database (7/2015–6/2021). Patients having ALPPS procedures, those having partial PLND and/or those having PLND for other than biliary tract cancers were excluded from the analysis. The study cohort included patients having liver resection and complete PLND for gallbladder cancer, ICC, or perihilar cholangiocarcinoma (primary cohort), as well as patients having liver resection without PLND (comparison group). Complete PLND was defined based on the following: being listed under the operative procedure in the Operative Note; documented definition, and skeletonization, of the HA, CBD, and PV, with isolation of the HA and CBD; *and* complete clearance of the lymphatic and fatty areolar tissues contained within the hepatoduodenal ligament, including *all* anterior and posterior compartments as defined in Figure 1B–E.

### 2.2. CAP-DI Surgical Technique

**Figure 2:**
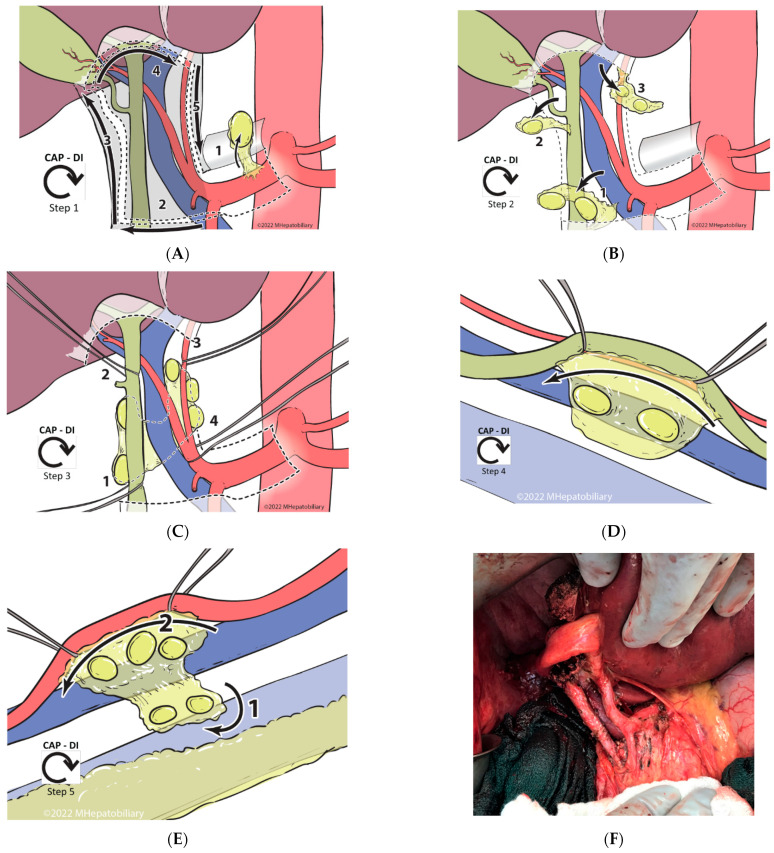
Technical steps of the Clockwise, Anterior-to-Posterior, Double-Isolation (CAP-DI) technique for portal lymph node dissection. (**A**). **Entry and release:** During this step, the peritoneal layer is entered above the hepatic artery lymph node, which is excised (1). This entry point is followed to release the peritoneal layer in a ***clockwise*** manner; starting across from the patient’s right (2), then cephalad along the bile duct (3), and subsequently across the hilum to the left side (4), to end caudally towards the entry point by following the left and proper hepatic arteries (5). (**B**). **Clockwise anterior lymph node dissection:** Following a clockwise approach and starting at the most inferior end of the hepatoduodenal ligament, the fatty areolar and lymphatic tissue in substation 2 between the proper hepatic artery and the common bile duct, and immediately anterior to the portal vein, is removed (1). Often it is necessary to sacrifice the right gastric vessels to retrieve the tissue in this location. While dissecting tissue off the surface of the portal vein, care must be taken to avoid injury to venous tributaries entering the anterior surface of the portal vein (i.e., coronary vein). Similarly, while it is important to excise all the tissue between the named structures, one should minimize dissection on the medial side of the bile duct to avoid injury to the 9 o’clock vessel. Subsequently, substation 3 is dissected along the cystic duct and within Calot’s triangle (2). The cystic duct, artery, and bile duct must be clearly identified and preserved while the lymph node dissection is performed. Upon completion of this step, a standard cholecystectomy is performed. Lastly, all lymphatic/fatty areolar tissue across the hepatic hilum is dissected anterior to the hepatic ducts and excised (3). It is important to not extend the dissection posterior to the ducts as this will increase the risk of injury to the duct and/or arteries, particularly in the setting of aberrant anatomy. (**C**). **Clockwise double isolation (2 structures at 2 sites):** Upon completion of the anterior lymph node dissection, the named structures should become clearly visible and accessible. Starting caudally and on the right, the distal common bile duct is dissected and encircled using a vessel loop. It is critical to have a direct view of the portal vein as this is being performed to avoid injury to its anterior and/or lateral surface (1). Subsequently, with the aid of the vessel loop to retract the bile duct anteriorly, the hepatic duct is dissected and encircled following the same precautions (2). Next, the left hepatic artery is identified and dissected at the distal level close to the umbilical fissure. In doing this, it is important to carefully dissect between the lymphatic tissue and the artery, and to have the distal portal vein in view to avoid injury (3). Lastly, the proper hepatic artery (just proximal to the take-off of the right hepatic artery) is identified and dissected. Note: the space between the gastroduodenal artery and the right hepatic artery is short. If the right gastric artery was not previously sacrificed, it may still need to be transected to allow adequate dissection and safe isolation of the proper hepatic artery; however, at this point, it can often be spared (4). (**D**,**E**) **Clockwise posterior lymph node dissection: Starting on the right side, the hepatic and bile duct are retracted anteriorly and medially to expose the plane between the posterolateral duct and the lymphatic tissue in the portocaval area.** This area is dissected cephalad until the lymph nodes/lymphatic tissue falls posteriorly off the duct. Using the left hand to expose, hold and retract the lymphatic tissue laterally, and a vein retractor to retract the duct and portal vein medially, the lymphatic tissue is dissected off from the posterolateral wall of the portal vein. Note that there is usually a portal tributary entering this side of the main portal vein, and this typically needs to be controlled, transected, and ligated prior to complete release of the lymphatic tissue from the vein. Lastly, the right portocaval lymph nodes are swept to the left side of the patient through the foramen of Winslow (2D). We then turn our attention to the left side of the hepatoduodenal ligament. The proper and left hepatic arteries are retracted anteriorly and laterally to allow exposure of the underlying lymph nodes/lymphatic tissue. With clear exposure of the distal portion of the main portal vein, the lymphatic tissue is dissected off the arteries, working cephalad to caudal until the common hepatic artery is reached. Using the right hand, the lymphatic tissue swept from the right hand is pulled to the left, allowing for clear delineation of the plane holding the residual tissue, which is then transected and excised (**E**). (**F**). Intraoperative photograph illustrating the results after complete PLND using a **CAP-DI** approach.

The goal of the ***CAP-DI*** approach is the removal of all node-containing tissue within the region in a standardized and reproducible manner, guided by the anatomic lymphatic substations located in the anterior and posterior compartments—defined by a coronal plane to the right and left of the anterior surface of the PV (Figure 1B–E). After complete Kocherization of the duodenum, and sampling of aortocaval and superior pancreatoduodenal lymph nodes (to exclude distant metastatic disease), a formal ***CAP-DI* PLND** is performed. For details of each step, please refer to Figure 2A–E. In short, the principle involves entry at the CHA lymph node substation with clockwise release of the peritoneal envelope around the hepatoduodenal ligament (Figure 2A), followed by clockwise removal of anterior lymphatic substations (Figure 2B). This leads to identification of the CBD, common, proper and left/right hepatic arteries, and the PV. Double isolation is then performed (2 structures at 2 different sites) in a clockwise manner using vessel loops around the distal CBD and the common hepatic duct, followed by isolation of the left hepatic artery and the proper hepatic artery (Figure 2C). With safe control of these structures, this step facilitates anterior retraction to enter the posterior compartment, where the right portocaval lymph nodes are first dissected off of the CBD/CHD and the right side of the PV, then swept across to the left side (Figure 2D). This is followed by anterolateral retraction of the proper and left hepatic arteries, and final dissection of the left periportal lymph nodes with en-bloc excision of the swept, right posterior nodal tissue (Figure 2E). Upon finishing and completely clearing all lymphatic and fatty areolar tissue in the hepatoduodenal area (Figure 2F), the PLND specimen is sent to pathology, separate from the liver specimen or other resected tissues. PLND yield in the pathology report is carefully reviewed; for those which yield < 6 lymph nodes, a pathology audit with re-review of the tissue is routinely performed.

### 2.3. Primary and Secondary Outcomes

The primary outcome was lymph node yield (total number of nodes retrieved during complete PLND). All pathology reports were reviewed to confirm that lymph nodes were obtained from the PLND portion of the operation. Predictors of ≥6 lymph node yield were subsequently identified. Secondary outcomes included intraoperative measures (operative time, operative blood loss, need for intraoperative transfusion) and postoperative outcomes (length of stay, 30-day grade III/IV complication, need for reoperation, need for unplanned readmission, bile leak, post-hepatectomy liver failure, and 90-day mortality). Secondary outcomes were identified and compared to the comparison group.

### 2.4. Statistical Analysis

Descriptive statistics are presented as: frequency, for categorical variables; and median with interquartile range (IQR), for continuous variables. Univariate analyses were performed using Chi-square, Fisher’s exact test or Wilcoxon rank-sum test, as appropriate. Exact logistic regression was used, when appropriate, given sample size to assess for factors associated with retrieval of ≥6 nodes. For secondary outcomes, multivariable analyses adjusting for operative factors—hepatectomy extent, bile duct resection, vascular resection, and visceral resection—were performed using logistic regression for binary outcomes, and linear regression for continuous outcomes. All statistical analyses were performed using Stata version 14 [20]. This study was approved by the Institutional Review Board at Moffitt Cancer Center and individual patient consent was waived.

## 3. Results

### 3.1. Study Cohort

In total, there were 534 patients included in the study; 71 patients having complete PLND for BTC diagnosis and 463 patients having hepatectomy without PLND. Cohort selection is displayed in Supplementary Figure S1. Baseline characteristics are shown in Table 1. Thirty-six cases (51%) were performed for gallbladder cancer, 24 (34%) for intrahepatic cholangiocarcinoma, and 11 (16%) for perihilar cholangiocarcinoma. The median age was 69 years (IQR 61–75) and the majority (*n* = 41, 58%) were female. Previous abdominal surgery was common in the cohort (*n* = 48, 68%) but previous liver surgery was not (*n* = 1, 1%). History of NASH was common (*n* = 26, 37%). Major hepatectomy was performed in 43 cases (61%), bile duct resection in 15 (21%) and vascular resection in 6 (9%).

### 3.2. Yield of Complete PLND Using the CAP-DI Technique and Predictors of ≥6 Node Yield

The median number of lymph nodes retrieved was 5 (IQR 3–8, range 0–17). Pathology showed fibroadipose tissue only with no identifiable lymph nodes in 2 cases. Thirty-three of 71 (47%) had at least 6 nodes retrieved. The distribution of lymph node yield is displayed in Supplementary Figure S2. 

Variables considered to have a plausible impact on lymph node yield were age, body mass index, receipt of preoperative chemotherapy, primary tumor site, previous abdominal surgery, preoperative portal vein embolization, and significant intraoperative blood loss (Table 2). Previous liver surgery was not assessed given that there was only one case. Among these variables, older age was found to be associated with a lower likelihood of ≥6 node PLND yield (*p* = 0.032). Among patients < 50 years in age (*n* = 4), the median yield was 12 nodes and ≥6 nodes were retrieved in all cases (*n* = 4/4, 100%). The median yield was 6 nodes for patients 50–69 years in age, and ≥6 nodes were retrieved in 51.5% (*n* = 17/33) of cases. The median yield was 5 nodes for patients ≥ 70 years in age and ≥6 nodes were retrieved in 35.3% (*n* = 12/34) cases. The association between older age and lower PLND yield remained significant when treating both variables as continuous (*p* = 0.014). A yield of ≥6 nodes was more common for gallbladder cancer (*n* = 17/36, 47%) and intrahepatic cholangiocarcinoma (*n* = 13/24, 54%) than for perihilar cholangiocarcinoma (*n* = 3/11, 27%), but this was not statistically significant (*p* = 0.33). No significant association between other potential predictors and PLND yield was identified (Table 2). The association between older age and lower yield remained significant in bivariate analyses using exact logistic regression when considering the other covariates individually (*p* < 0.05 for all). No models containing three or more variables are presented due to sample size considerations.

### 3.3. Intraoperative Characteristics and Postoperative Outcomes

Secondary outcomes associated with performance of PLND, including intraoperative characteristics and postoperative outcomes, were assessed by contrasting the primary PLND cohort (*n* = 71) to the comparison group not undergoing PLND (*n* = 463). These are displayed in Table 3. In univariate analyses, the PLND group had higher intraoperative blood loss (mean 337 mL versus 293 mL, *p* = 0.055), need for transfusion (13% versus 7%, *p* = 0.062), and operative time (330 min versus 260 min); as well as increased rate of 30-day Grade III/IV postoperative complication (17% versus 8%, *p* = 0.003), postoperative bile leak (14% versus 3%, *p* < 0.001), and 90-day postoperative mortality (4% versus 1%, *p* = 0.02). However, when adjusted for operative factors, including extent of hepatectomy (major versus minor), bile duct resection, vascular resection, and visceral resection, only longer operative time remained significantly associated with performance of PLND (+46.4 min after adjustment, *p* = 0.001). Additional models with adjustment for factors significantly different between the primary cohort and comparison group (Table 1) were explored with the consistent finding that operative time was longer for the PLND cohort without other significant differences in intraoperative or postoperative outcomes. 

## 4. Discussion

Complete PLND is the current standard for oncologic resection of BTCs for adequate surgical staging [1,2]. However, registry data show that it is performed infrequently and often yields less than the recommended 6 lymph nodes [11,13,14,15,16]. In the current study, we describe results following the ***CAP-DI*** technique for PLND; a stepwise, reproducible approach. In this method, nodal-containing tissue of the hepatoduodenal ligament is dissected in a clockwise pattern, first on the anterior aspect of nodal basin with double isolation of critical structures, followed by a posterior nodal compartment dissection. The ***CAP-DI*** technique is intended to be deliberate and methodical, and therefore suitable as a teach-and-train technique. Using the ***CAP-DI*** technique, the median PLND yield was 5 lymph nodes and ≥6 nodes were obtained in 47% of cases. Older age was found to be associated with a lower yield of PLND, with patients age ≥ 70 years having 6 or more nodes obtained 35% of the time. After adjustment for operative factors, ***CAP-DI*** added an average of 46 min of operative time without an associated increase in intraoperative or postoperative complications when contrasted with the comparison group not undergoing PLND.

The current finding that the median yield following PLND falls below the recommended threshold of 6 nodes is consistent with other published data. In analyses using registry data from the National Cancer Data Base (NCDB) or SEER, it has been reported that obtaining 6 nodes occurs infrequently, approximately 25% of the time [11,13,14,15,16]. These studies are informative about national practice patterns for performing PLND and show that there is much room for improvement toward performing nodal staging for all patients as recommended by the AJCC staging criteria. However, they lack granularity as it is impossible to know if the nodal yield is due to an incomplete dissection being incorrectly documented as a PLND, or if, in fact, the true contents of the regional nodal basin frequently harbor fewer than 6 nodes. In institutional series, yield is frequently higher yet still falls below the recommended threshold of 6 nodes. For example, in the analysis from Memorial Sloan Kettering which was influential in defining the 6-node threshold, the median nodal yield was 3 [10]. Leigh and colleagues similarly found that their yield of lymphadenectomy, when performed for gallbladder cancer, met the recommended threshold only 20% of the time, and no nodes were found upon pathology in 9% of cases [18]. The finding in the present study that younger patients are more likely to have ≥6 nodes retrieved is consistent with reports using the NCDB, which also found that provider-level factors, including treatment at an academic institution and private insurance provider, are associated with higher nodal yield [11,14]. While these latter factors identified through registry data likely represent a surrogate of quality of care, older age may indeed be a biologic factor associated with lower nodal yield. Supporting this is the consistent pattern seen in colorectal and gastric cancer [21,22]. Additionally, according to the NCDB, an open surgical approach is associated with a higher yield compared to laparoscopic approaches [17].

While the prognostic benefit of removing 6 nodes for more accurate staging appears clear, it is debatable whether aggressively removing nodes beyond the regional basin to meet this threshold is warranted. Involvement of nodes beyond the basin portends a poor prognosis on the spectrum of metastatic disease [1]. Consistent with this, in the case of gallbladder cancer, expert consensus has recommended biopsy of these nodes for a tailored surgical approach, but with formal dissection limited to the hepatoduodenal ligament [23]. Given the current study’s findings, taken in context with other reports, one might not expect to find a minimum of 6 nodes within the confines of the regional nodal basin. While all attempts should be made for a thorough regional lymphadenectomy to optimize staging for prognostication and consideration of adjuvant therapies, use of the 6-node threshold as a quality metric may not be appropriate.

While nomenclature varies, the goal of the ***CAP-DI*** technique is to provide a reproducible method of removing the nodes frequently referred to as 12a, 12b, 12c, 12p, and 12h, as well as in stations 13 and 14 in the Japanese classification system [19,24]. Detailed anatomic and cadaver studies have described lymphatic drainage from the gallbladder and liver through these nodes, thus making them the target of regional dissection [25,26,27,28]. Sato and colleagues draw particular attention to the importance of the node of the epiploic foramen of Rouviere as well as the periportal node, both of which should be accessible during the anterior phase at step 2B in the ***CAP-DI*** approach [27]. There are few published technical descriptions of PLND. Pandey provides one such description, which differs from the ***CAP-DI*** method in that retroportal dissection is undertaken early, and the general focus is on individual structures separately rather than a clockwise geographic approach [29]. We consider that a strength of the ***CAP-DI*** approach is its reproducibility, owing to an overarching perspective broken into sequential steps. Regardless of the specific technique, the goal of PLND should be the thorough removal of all lymph node-containing tissue in the hepatoduodenal ligament. The ***CAP-DI*** approach may be a reproducible method to achieve this goal for those who do not perform PLND with great frequency.

This analysis was retrospective in nature with the inherent limitations as such, and there are other important limitations which should be noted. Statistical analysis was limited by the relatively small sample size of the primary study cohort. Published registry data describing yield and predictors of PLND are obviously much stronger in statistical power than the current study. However, as noted above, those data lack granularity, so the motivation for the current study was, in part, to examine these outcomes with the advantage of access to primary data; indeed, we found our institutional experience had a higher nodal yield than typically reported in registry data, but which, consistent with other institutional reports, still frequently fell below the 6-node threshold. The current study does not address oncologic outcomes associated with greater PLND as this is better addressed with larger datasets. Furthermore, this study contains a mix of BTCs as the major focus was on the technical aspects and immediate outcomes of PLND; this also limits any conclusions about oncologic outcomes.

## 5. Conclusions

PLND is the current standard for oncologic resection for BTCs, yet it is performed infrequently and often yields less than the recommended 6 lymph nodes. The current report provides a technical description of the ***CAP-DI*** method of PLND, which is easily reproducible, well aligned with the corresponding surgical anatomy of the nodal substations located in the hepatoduodenal ligament, and allows for better adherence to surgical nodal staging of BTCs. Performance of PLND using this technique was associated with a longer average operating time of 46 min without differences in intraoperative or postoperative outcomes compared to a comparison group. Older age appears to be associated with a lower PLND yield, frequently falling below the recommended threshold of 6 nodes, consistent with other published data. While complete PLND with a high yield of lymph nodes retrieved is encouraged and associated with better staging, rigid expectations of retrieving a minimum of 6 nodes may be unrealistic; therefore the use of this threshold as a *quality metric* should be reconsidered.

## Figures and Tables

**Table 1:** Characteristics of Study Cohort.

	Complete Portal Lymphadenectomy *n* = 71	Comparison Group *n* = 463	
Patient and Clinical Factors	*n* (%)	*n* (%)	*p*
Age, years [median (IQR)]	69 (61–75)	62 (53–70)	<0.001
Female	41 (58)	237 (51)	0.30
Modified Charlson Score			
0	37 (52)	293 (63)	0.27
1	21 (30)	109 (24)	
2	9 (13)	36 (8)	
3+	4 (6)	25 (5)	
BMI > 30	32 (45)	160 (35)	0.09
Preoperative chemotherapy	16 (23)	260 (56)	<0.001
Previous abdominal surgery	48 (68)	326 (71)	0.61
Repeat liver surgery	1 (1)	39 (8)	0.037
**Liver Function**			
Cirrhosis	4 (6)	21 (5)	0.76
NASH	26 (37)	37 (8)	<0.001
Hepatitis B or C	7 (10)	23 (5)	0.096
ALBI score			0.58
Grade 1	62 (87)	424 (92)	
Grade 2	9 (13)	37 (8)	
Grade 3	0 (0)	2 (0.4)	
**Tumor Factors**			
Diagnosis			<0.001
Gallbladder cancer	36 (51)	0 (0)	
Intrahepatic cholangiocarcinoma	24 (34)	4 (1)	
Perihilar cholangiocarcinoma	11 (16)	0 (0)	
Hepatocellular carcinoma	--	45 (10)	
Colorectal liver metastases	--	208 (45)	
NET liver metastases	--	120 (26)	
Other	--	86 (19)	
**Operative Factors**			
Extent of Hepatectomy			0.21
Major hepatectomy	27 (39)	144 (31)	
Minor hepatectomy	43 (61)	319 (69)	
Bile Duct Resection	15 (21)	3 (1)	<0.001
Vascular Reconstruction	6 (9)	6 (1)	0.002
Concurrent Visceral Resection	4 (6)	112 (24)	<0.001

**Table 2:** Intraoperative and postoperative outcomes of PLND.

	Complete Portal Lymphadenectomy *n* = 71	Comparison Group *n* = 463	Multivariable *		
			*p*	Point Estimate (95% CI)	*p*
**Intraoperative Characteristics**					
Estimated Blood Loss, mL	200 (150–400)	200 (100–400)	0.055	7.8 (−81.8–97.5)	0.86
Intraoperative Transfusion	9 (13)	30 (7)	0.062	1.49 (0.51–4.34)	0.46
Operative Time, minutes	311 (225–412)	239 (180–321)	<0.001	46.4 (19.5–73.4)	0.001
**Postoperative Outcomes**					
Length of stay, days	5 (4–7)	5 (4–7)	0.85		
Grade III–IV Complication, 90-day	12 (17)	39 (8)	0.003	1.14 (0.39–3.36)	0.81
Post-hepatectomy Liver Failure			0.45		
Grade A	2 (3)	18 (4)			
Grade B	2 (3)	5 (1)			
Grade C	1 (1)	2 (0.4)			
Postoperative Bile Leak **			<0.001		
Grade A	4 (6)	6 (1)		2.46 (0.58–8.23)	0.24
Grade B	6 (9)	6 (1)			
Grade C	0 (0)	1 (0.2)			
Unplanned reoperation, 90-day	2 (3)				
Readmission, 90-day	7 (10)	47 (10)	0.94		
Mortality, 90-day	3 (4)	4 (1)	0.02	0.71 (0.02–13.2)	1.0

Median (IQR) is shown for continuous variables. Models were performed using linear regression for continuous outcomes (estimated blood loss, operative time), logistic regression (transfusion, grade III–IV complication), and exact logistic regression for outcomes with few events (bile leak, mortality). * Adjusted for hepatectomy extent, bile duct resection, vascular resection, and visceral resection. ** Bile leak of any grade was collapsed to a single binary variable for multivariable analysis.

**Table 3:** Predictors of ≥6 nodes retrieved with complete portal lymphadenectomy (*n* = 71).

	Median (IQR)	0–5 Nodes	≥6 Nodes	*p*
Overall Cohort	5 (3–8)	38 (54)	33 (47)	--
Age				0.032
<50	12 (10–14) *	0 (0)	4 (12)	
50–69	6 (4–8)	16 (42)	17 (52)	
≥70	5 (3–6)	22 (58)	12 (36)	
BMI ≥ 30	6 (4–9)	15 (40)	17 (52)	0.31
Preoperative chemotherapy	6 (3–10)	7 (18)	9 (27)	0.37
Tumor site				0.33
Gallbladder	5 (4–8)	19 (50)	17 (52)	
Intrahepatic cholangiocarcinoma	6 (4–9)	11 (29)	13 (39)	
Perihilar cholangiocarcinoma	4 (1–8)	8 (21)	3 (9)	
Previous abdominal surgery	5 (4–8)	27 (71)	21 (64)	0.51
Preoperative PVE	4 (3–8)	5 (15)	3 (10)	0.71
EBL > 1000 or transfusion	4.5 (4–7)	6 (16)	4 (12)	0.74

* Number of nodes retrieved were 6, 10, 14, and 17.

## Data Availability

The data presented in this study are available on reasonable request from the corresponding author.

## Supplementary Materials

The following supporting information can be downloaded at: https://www.mdpi.com/article/10.3390/cancers14235770/s1, Figure S1: STROBE cohort selection; Figure S2: Yield of portal lymphadenectomy. Median number of nodes retrieved was 5.
