# Supplementary material for: Clockwise Anterior-to-Posterior—Double Isolation (CAP-DI) Approach for Portal Lymphadenectomy in Biliary Tract Cancer: Technique, Yield, and Outcomes
Source: Cancers (Basel). 2022 Nov 24;14(23):5770. doi: 10.3390/cancers14235770 (PMC9738406; doi:10.3390/cancers14235770)
Supplement: Supplementary file 1 [file cancers-14-05770-s001.zip › cancers-2020462-supplementary.pdf]

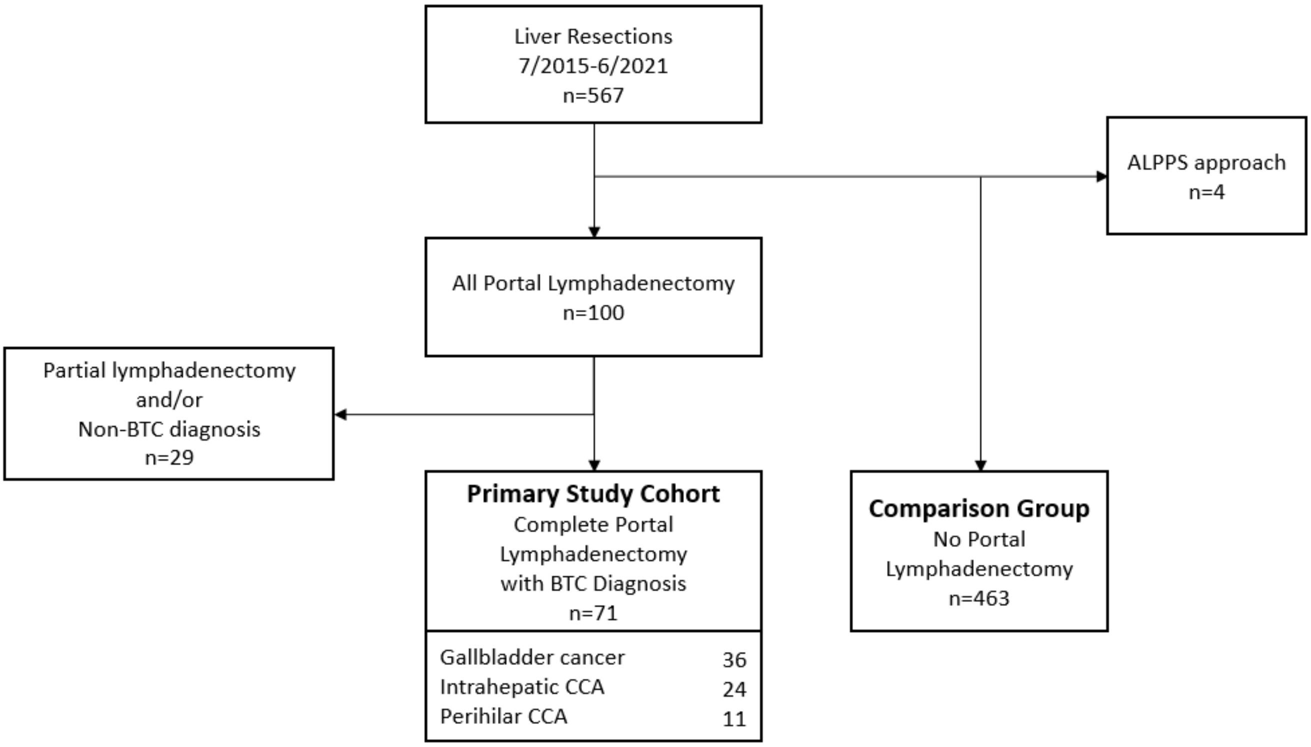


**Supplemental Figure S1.** STROBE cohort selection

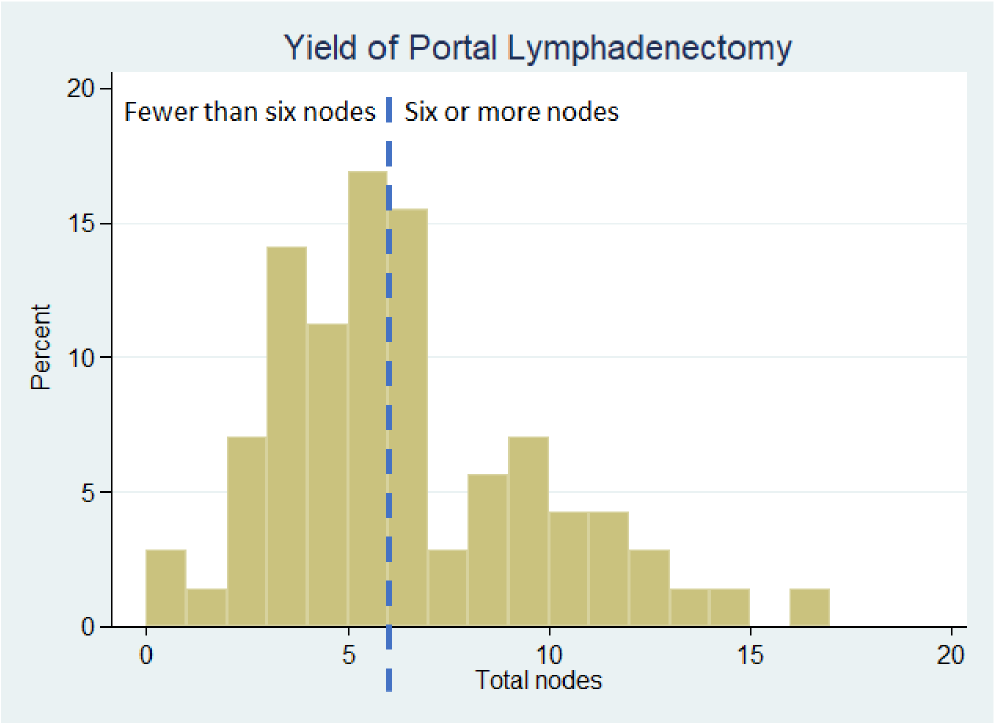


**Supplemental Figure S2.** Yield of portal lymphadenectomy. Median number of nodes retrieved was 5
